# Cancer incidence in Germany attributable to human papillomavirus in 2013

**DOI:** 10.1186/s12885-017-3678-6

**Published:** 2017-10-16

**Authors:** Nina Buttmann-Schweiger, Yvonne Deleré, Stefanie J. Klug, Klaus Kraywinkel

**Affiliations:** 1 Department of Epidemiology and Health Monitoring, Robert Koch-Institut, Berlin, Germany; 2 General practitioner, Rudower Str. 60, 12524 Berlin, Germany; 3 Cancer Epidemiology, University Cancer Center Dresden, University Hospital, Technische Universität Dresden, Dresden, Germany; 4 Epidemiology, Department for Sport and Health Sciences, Technical University of Munich, Munich, Germany; 5 Robert Koch-Institut, Department of Epidemiology and Health Monitoring, German Centre for Cancer Registry Data, General Pape-Straße 62-68, 12101 Berlin, Germany

**Keywords:** Human papillomavirus, Cancer, Epidemiology, Registry data, Germany

## Abstract

**Background:**

It is estimated that a total of 120,000 new cancer cases in men and in women in more developed countries could be avoided if exposure to HPV was prevented. We used the nationwide pool of German population-based cancer registry data to estimate the burden of HPV-attributable cancer in this population for the year 2013.

**Methods:**

Incident cases of cervical cancer, squamous cell carcinoma of the anus, oropharynx (OP), as well as of the vulva, vagina and penis were classified as potentially HPV-associated and identified from the nationwide cancer registry data-pool. We calculated the incidence and proportions of cancer with potentially HPV-associated morphologies. Estimation of the HPV-attributable incidence was based on prevalence-estimates of viral DNA in tumor cells in the respective sites, as provided from the international literature.

**Results:**

From the overall 15,936 incident cases of anogenital and OP cancers in 2013, 6239 female and 1358 male cancer cases were estimated to be attributable to HPV. The majority of HPV-attributable cases were contributed by cervical cancer (70.9% of female cancers) and oropharyngeal cancer (46.9% of male cancers).

**Conclusions:**

Even if most HPV-attributable cases were contributed by cervical cancer, anogenital cancer at sites other than the cervix, and oropharyngeal cancer substantially contribute to the burden of HPV-associated cancer. Our nationwide cancer registry data-analyses provide the baseline for long-term population-based monitoring of vaccination-effects on cancer incidence in Germany.

## Background

Persistent infection with Human Papillomavirus (HPV) is considered a necessary cause in the etiology of virtually all cases of invasive cervical cancer [1]. In Germany, 4600 women were diagnosed with invasive cervical cancer in 2012 [2]. Increasing evidence further suggests HPV to be causally related to squamous cell carcinomas of another five cancer sites: the penis, vulva, vagina, anus and subsites of the oropharynx. It is estimated that a total of 120,000 new cancer cases in men and in women in more developed countries could be avoided if exposure to HPV was prevented [3].

Monitoring of HPV-associated disease remains crucial as two prophylactic HPV-vaccines have been licensed in Germany in 2006 and are recommended since 2007 by the German Standing Committee on Vaccination (STIKO). In 2014, the recommended age for vaccinating girls was lowered from 12–17 years to 9–14 years [4]. With the introduction of the HPV-vaccine and implementation in the German vaccination schedule, cervical cancer might become a largely preventable disease. The current vaccines are highly effective in preventing infections with HPV-types 16 and 18, the types that cause most cervical and anal cancers as well as anogenital pre-cancers [5]. High vaccine efficacy in protection against HPV-16/18 infections in oral sites has been demonstrated [6]. A newly developed nonavalent HPV-vaccine was recently licensed in the European Union (EU), which further prevents infection with HPV-31, 33, 45, 52, and 58 [7] that may cause about 15% of cervical cancers [8].

We used the nationwide pool of German population-based cancer registry data to assess the baseline incidence of HPV-associated cancer in our population for the year 2013, yet before quantifiable effects from HPV-vaccination on cancer incidence can be anticipated. Thus, our analyses provide the basis for a continuous monitoring of the effects of HPV-vaccination on cancer incidence at the population-level. This also applies to HPV-associated cancer in men, as a positive effect from vaccinating girls on HPV-related outcomes in men is expected [9]. Potential future discussions on vaccinating boys against HPV might be triggered by the findings of this study.

## Methods

### Classification of potentially HPV-associated cancer and estimation of the HPV-attributable fraction

Cases of cervical cancer with epithelial, squamous cell, basal and transitional cell origin, adenocarcinomas and cystic carcinomas as well as mixed tumors; squamous carcinoma of the anus and oropharynx, the vulva, vagina and penis were classified as potentially HPV-associated carcinoma (Table 1). The International Agency for Research on Cancer (IARC) established a causal role for human papillomavirus in a subset of these carcinoma, defining HPV-associated sites [10].

**Table 1:** HPV-associated cancer sites and morphologic specification

Site	ICD-O-3 topography code	Potentially HPV-associated morphology	ICD-O-3-codes
Cervix	C53	All carcinoma (except for melanoma, mesothelioma, Kaposi sarcoma, lymphoma, leukemia and other myeloproliferative malignant disorders)	8010–8671, 8940–8941
Penis	C60	Squamous cell carcinoma (SCC)	8050–8084
Vulva	C51	SCC	8050–8084
Vagina	C52	SCC	8050–8084
Anus	C21 (incl. C20 with ICD-O-3 8050–8084, 8120–8131)	SCC	8050–8084
Oropharynx, incl. base of tongue and tonsils	C01, C02.4–02.9, C05.1–05.9, C09.0–09.9, C10.0–10.9, C14.0-C14.8	SCC	8050–8084

Pathophysiological knowledge and epidemiologic evidence strongly suggest that the presence of HPV-infection in tumor material is sufficient to infer that HPV-infection caused the cancer. Estimation of the population attributable fraction (PAF) due to HPV was therefore based on the prevalence of viral DNA in squamous cell carcinoma (SCC) cells in the respective sites, as provided from the international literature, preferably from most recent metaanalyses and large tissue sample studies [1, 11–15]. From the vaginal, penile and anal cancer studies, the HPV-DNA prevalence in European invasive SCC was chosen to derive the HPV-attributional fractions [12–14]. In oropharyngeal cancer, the HPV-attributional fraction was more specifically derived from the prevalence of the combination of HPV-DNA positivity, mRNA positivity or positivity for a cell surrogate marker of HPV-induced carcinogenic transformation (p16^INK4a^ overexpression) - a validated and widely used HPV-detection algorithm [16]. The oropharynx included the base of tongue, lingual tonsil, overlapping lesions of tongue, tongue not otherwise specified, soft palate, uvula, overlapping lesions of palate and palate not otherwise specified, the tonsils, oropharynx, and other ill-defined sites in lip, oral cavity and pharynx including Waldeyer ring. For the HPV-attributable fraction in vulvar SCC, we used the crude European HPV-prevalence from HPV-DNA positive/p16^INK4a^ overexpressive keratinizing or warty-basaloid vulvar SCC [11].

Namely, all cases of cervical cancer and 88–90% of anal carcinoma with HPV-associated morphologies were considered HPV-attributable. An HPV-attributable proportion of 32% was assumed in penile cancer with HPV-associated morphologies, whereas 81% of vaginal, 18% of vulvar, and 16% of OP cancer with potentially HPV-associated morphologies were considered HPV-attributable (Table 2).

**Table 2:** Incident anogenital and oropharyngeal cancer cases and estimation of HPV-attributable cancer in Germany, 2013

Cancer site	Incident cases independent of morphology	Incident cases of HPV-associated morphology	(Proportion estimated)^a^	HPV-attributable fraction PAF	Reference	Estimated HPV-attributable incident cases	(Proportion of overall anogenital and oropharyngeal cancer)
Cervix	4458	4422	(7.1%)	100%	[1]	4422	(70.9%)
Vulva	3015	2596	(4.0%)	18%	[11]	467	(7.5%)
Vagina	386	275	(9.6%)	81%	[13]	223	(3.6%)
Female anus	1142	1030	(5.0%)	90%	[14]	927	(14.9%)
Female oropharynx	1371	1249	(7.5%)	16%	[15]	200	(3.2%)
Female overall anogenital & oropharynx	10,372	9573	(6.1%)			6239	(100%)
Penis	757	704	(6.3%)	32%	[12]	225	(16.6%)
Male anus	653	564	(3.9%)	88%	[14]	496	(36.5%)
Male oropharynx	4154	3978	(5.8%)	16%	[15]	636	(46.9%)
Male overall anogenital & oropharynx	5564	5246	(5.7%)			1358	(100%)

^a^ proportion of estimated cases with HPV-associated morphology that were not coded as such, but redistributed from cases of not otherwise specified morphology

### Monitoring of HPV-associated morphology in cancer registry data

Population-based cancer registration in Germany is organized by the 16 federal states, six of them merged to a joint registry for East Germany (including Berlin). Continuous registration has commenced in 1970 in the Saarland, but nationwide coverage has not been reached before 2009, when the population-based cancer registry of Baden-Württemberg was implemented. According to our current nationwide estimation, the degree of cancer registration across Germany is high, and 96% (463,611 cases) of the estimated 482,473 new cancer cases in 2013 have actually been recorded in the registries (data not shown). For our analyses, we retrieved incident cases of cervical cancer, penile cancer, vulvar cancer, vaginal cancer, anal cancer, and oropharyngeal cancer from the nationwide data pool for 2013. Site, behavior and histology are coded according to the International Classification of Diseases for Oncology (ICD-O-3).

For the purpose of monitoring, we provide the number of newly diagnosed cases, proportions and rates of cancer with potentially HPV-associated morphologies. Morphology codes of potential HPV-association are shown in Table 1. To correct for missing information on further morphologic specification of cancer cases (ICD-O-3 group 800: neoplasms, not otherwise specified), we redistributed these cases according to the distribution of morphology codes among those cases with further specified morphology (by site, gender and 5-year-age-group), assuming the information on morphology was ‘missing at random’. Cases notified from death certificate only (DCO) represented the majority of morphologically not otherwise specified cases.

Incidence rates from 2013 were age-standardized to the old European standard population (ASIR). 95% confidence intervals of ASIR were calculated using the binomial approximation according to the recommendation of IARC [17]. Calculation of the 95% confidence intervals of crude age-specific rates was based on a Poisson-distribution of the cases.

## Results

In 2013, 5564 male incident cases and 10,372 female incident cases of invasive anogenital and oropharyngeal cancers were registered in Germany. Overall 9573 incident female cancer cases with potential HPV-associated site and morphology were extracted from the pooled data. Amongst them were 6239 incident cases considered to be attributable to HPV; 4422 cervical cancers (70.9% of all HPV-attributable female cancer cases), 927 anal cancers, 467 vulvar cancers, 223 vaginal cancers, and 200 oropharyngeal cancers. Among the 5246 incident male cancer cases with potential HPV-associated site and morphology, 1358 cases were attributed to HPV. Oropharyngeal cancer constituted the largest group (636 cases; 46.9% of all HPV-attributable male cancer cases) followed by anal cancer (496 cases) and penile cancer (225 cases). Table 2 provides the site-specific results and illustrates our calculations: for vaginal cancer, 386 incident cases were registered in 2013, of which 275 cases were of HPV-associated morphology. A proportion of 9.6% (26 cases) of the 275 cases with HPV-associated morphology were not coded as such, but redistributed from cases morphologically not otherwise specified. Applying the assumed PAF of 81% [13], 223 vaginal cancer cases were finally considered attributable to HPV.

Overall, a proportion of 1.6% of an estimated 482,473 new cancer cases in Germany in 2013 was considered HPV-attributable. The number of 9573 female cancer cases and 5246 male cancer cases with potential HPV-associated site and morphology assessed corresponds to approximately 3.1% (male: 2.1%; female: 4.2%) of the overall cancer burden in Germany in the same year. The proportion of incident cases with potentially HPV-associated morphology that were redistributed from cases morphologically not otherwise specified ranged between 3.9% (male anus) and 9.6% (vagina).

The most common cancers in the female and male population were cervical cancer in women and oropharyngeal cancer in men. Non-cervical anogenital cancer with HPV-associated morphology were rare in the German population. Anal cancer with an HPV-associated morphology was however more common in women than in men (Table 3).

**Table 3:** Age-standardized incidence rate (ASIR) of cancer in men and women with HPV-associated morphology, Germany 2013

	Men		Women	
	ASIR	95% CI	ASIR	95% CI
Overall anogenital	2.3	2.2–2.4	14.8	14.4–15.1
Cervix	–	–	8.9	8.6–9.2
Penis	1.2	1.1–1.3	–	–
Vulva	–	–	3.7	3.6–3.9
Vagina	–	–	0.4	0.3–0.4
Anus	1.1	1.0–1.2	1.7	1.6–1.8
Oropharynx	7.6	7.4–7.9	2.2	2.0–2.3

Age-standardized incidence rates (European standard population) and corresponding 95% confidence intervals (95%CI) per 100,000 population; HPV-associated morphology codes (ICD-O-3): 8010–8671, 8940–8941 (cervix), 8050–8084 (penis, vulva, vagina, anus, oropharynx)

**Fig. 1:**
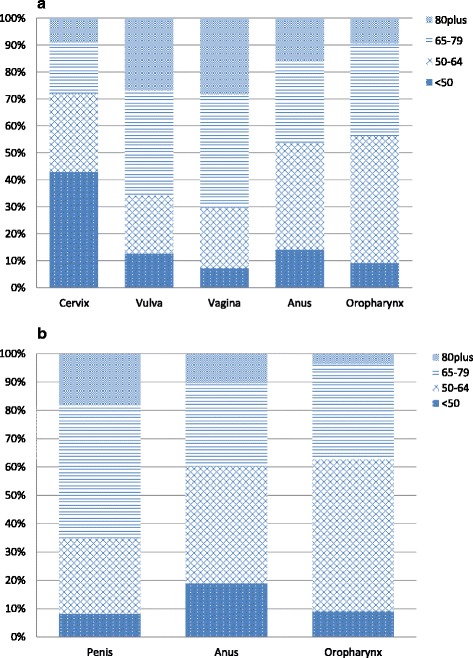
Distribution of age-groups in female (**a**) and male (**b**) cancer with HPV-associated morphology, Germany 2013

The age-distribution largely differed by site (Fig. 1a and b). The largest proportion of patients diagnosed below the age of 50 years was seen among cervical cancer cases (42.9%). Almost half of all anal cancer cases and female oropharyngeal cancer patients were 50–64 years of age (39.6% of female anal cancer patients, 41.2% of male anal cancer patients, and 47.0% of female oropharyngeal cancer patients respectively). Penile, vulvar and vaginal cancer patients were comparably older at diagnosis. The age distribution of male oropharyngeal cancer patients closely resembled the one of female oropharyngeal cancer patients (Fig. 1b): 9.1% of men were younger than 50 years (in females: 9.3%), 53.5% were aged 50 to 64 years (in females: 47.0%), 33.7% were aged 65 years to 79 years (in females 34.5%) and 3.7% were 80 years or older (in females: 9.4%).

Correspondingly, crude incidence rates (IR) of cervical cancer were highest in the age groups of 35–49 years and 50–64 years (16.5 per 100,000 and 14.8 per 100,000, respectively) and hereafter declined (Table 4). IR of vulvar, vaginal, anal, and penile cancer increased with increasing age. For oropharyngeal cancer, incidence rates peaked at ages 50–64 years both in men (IR: 24.8 per 100,000) and in women (IR: 6.7 per 100,000) and hereafter declined (Table 4).

**Table 4:** Age-specific incidence-rates (95% confidence intervals) of female and male cancer with HPV-associated morphology by age-groups, Germany 2013

		35–49 years		50–64 years		65–79 years		≥80 years	
		IR	95% CI	IR	95% CI	IR	95% CI	IR	95% CI
Women	Cervix	16.5	15.6–17.4	14.8	14.0–15.6	12.4	11.6–13.3	14.1	12.8–15.6
	Vulva	3.3	2.9–3.7	6.4	5.9–7.0	15.0	14.1–16.0	24.3	22.6–26.2
	Vagina	0.2	0.1–0.3	0.7	0.5–0.9	1.7	1.4–2.1	2.7	2.1–3.4
	Anus	1.5	1.3–1.8	4.7	4.2–5.2	4.7	4.2–5.2	5.8	4.9–6.7
	Oropharynx	1.3	1.1–1.5	6.7	6.2–7.3	6.4	5.8–7.1	4.1	3.4–4.9
Men	Penis	0.6	0.4–0.8	2.2	1.9–2.5	5.8	5.2–6.4	8.8	7.3–10.4
	Anus	1.1	0.9–1.4	2.7	2.4–3.1	2.9	2.5–3.4	4.0	3.1–5.2
	Oropharynx	4.0	3.6–4.5	24.8	23.8–25-9	23.4	22.1–24.7	10.0	8.5–11.8

Age-specific incidence rates (IR) and corresponding 95% confidence intervals (95%CI) per 100,000 population

## Discussion

Overall, the 6239 incident female HPV-attributable cancer cases and 1358 incident male HPV-attributable cancer cases relate to approximately 1.6% HPV-attributable incident cancer cases of the 482,473 new cancer cases in Germany in the same year.

Similar estimates have been reported in other populations of the western world [18, 19]. Despite methodological differences in selection of HPV-prevalence estimates, different contribution of cancers related to other risk factors (such as tobacco smoking or alcohol consumption) on the overall and site specific cancer burden, and potentially different degrees of completeness of cancer registry data, our estimates are comparable with recent 2010 figures from the United Kingdom (UK) (1.6%) [18], Australia (1.5%) [19], and the approximately 2.0% HPV-associated cancers in Europe, proposed in a recent synthesis on the global burden of infection-associated cancer [3]. The latter was based on the GLOBOCAN-database provided by IARC, which provides incidence figures by cancer site and country, but not further specified by morphology. Hence, our more conservative approach of applying the HPV-attributable fractions on cases that were of potential HPV-associated morphology only, resulted in somewhat lower estimates of HPV-related disease burden compared to the European estimates provided by DeMartel et al. [3].

An aspect that limits precision of our results is introduced by using HPV-attributable fractions from the international literature and their application to our national incidence.

Population-based cancer registries neither collect information on HPV in tumor tissue nor on other individual risk factors such as smoking. Hence, the proportion of HPV-attributable cases, which were derived mostly from recent metaanalyses or large case series, might be over- or underestimated. As prevalence of HPV is likely to vary by country, age, gender (in case of oropharyngeal and anal cancer) and time, the actual HPV-prevalence among cancer cases in Germany might deviate from the prevalence estimates we presumed. In oropharyngeal cancer, previous studies from Germany that were included in a recent metaanalysis [20] and one recent German study based on a clinical sample reported higher HPV-prevalence as the European estimate of 16% provided by Castellsague et al. [15, 21]. Differences in methods of HPV-testing usually hamper precise comparisons of HPV-prevalence over time and study population – an important advantage of the highly standardized study protocol and the single-centre HPV-DNA detection and genotyping in the study by Castellsagué et al. The authors further describe an increasing proportion of HPV-positive oropharyngeal cancer cases over time (worldwide): only about 10% of oropharyngeal cancer cases diagnosed in the 1990s were HPV-DNA-positive and mRNA-positive/p16-positive. In oropharyngeal cancer cases diagnosed in 2010–2012, one third was HPV-DNA-positive and mRNA-positive/p16-positive [15]. It is not clear if HPV-prevalence in the German population is increasing, but recent data indirectly suggests that the observed increase of OP-SCC in the younger population in countries with declining smoking prevalence might be explained by increasing HPV-prevalence [22–24]. Given the changing epidemiology of HPV-positive oropharyngeal cancer over time, our numbers might underestimate the actual HPV-attributable fraction. Rising HPV-prevalence may also partly explain the increasing trends in vulva cancer which we recently described for Germany [25].

The quality of epidemiological cancer registry data limits registry-based studies in general, and also needs to be considered in our study: An aspect of uncertainty is introduced by potentially incomplete registration of incident cases in some registries, leading to underestimation. However, nine German federal states had achieved sufficient data quality and completeness to be included in the IARC publication “Cancer in five continents (CI5)” for the years 2003–2007, which can be regarded as a reference for high quality registries [26]. Since 2009, incident cancer cases are registered nationwide and quality has well improved. A comparison of the estimated nationwide incidence indicates that we might have also underestimated the incidence of anogenital and oropharyngeal cancer by up to 5%. The relatively high proportion of cases notified from death certificate only (DCO) (3–10% by cancer site considered in our analysis) similarly suggests certain underreporting in German cancer registries. Another source of bias was introduced by missing information on morphologically not otherwise specified cancer in some cases: overall, 6.1% of morphologically HPV-associated cases in women and 5.7% in men were estimated due to missing information on morphology (including DCO).

In recent studies of penile, anal, vaginal and vulvar cancer, the histological term “warty” or “basaloid/warty” carcinoma has been used to characterize HPV-associated carcinoma [11–14]. Only recently, this term was introduced in the updated World Health Organization (WHO)-classification of tumours of the penis, and a new code (8054/3) for this entity was suggested [27]. The former code for “not otherwise specified keratinizing squamous cell carcinoma (8071/3 keratinizing SCC, NOS)” was omitted. At present, the cancer registry data based on ICD-O-3 classification does not yet provide this specific information. This limitation also applies to the issue of monitoring time trends at the other anogenital sites. The PAF for basaloid/warty cancer would be much higher than the PAFs we applied (e.g. an attributable fraction of 57% in warty/basaloid vulvar SCC compared to 18% in all invasive vulvar cancer [11]), however applied to fewer cases with HPV-associated morphology. Further morphology-, age- and site-specific analyses of pathology records are in preparation to verify our literature-based assumption on the HPV-attributable proportions in SCC.

Due to missing information on site specific HPV-prevalence in the general German population [28] our PAF-estimates are based upon HPV-prevalence in tumor tissue from international studies and therefore should be considered an approximation. Prevalence in tumor tissue might not necessarily be causally related to cancer development, especially if other risk factors are present. Tobacco-smoking constitutes another major risk factor for anogenital and oropharyngeal cancer, as well as immunodeficiency with HIV-positivity or immunosuppression following organ transplantation [29–32].

It should be mentioned, that our estimates only take invasive tumors into account, while especially for the cervix, a large number of high-grade dysplastic lesions contribute to the HPV-related disease burden, demanding invasive treatment. Similar to other published estimates on HPV-related disease burden, we did not consider these cases in our analyses.

HPV-associated anogenital cancer and oropharyngeal cancer substantially contribute to the burden of cancer. In the United States of America (US), cancer with HPV-associated site and morphology account for 2% of male cancer cases and 3.3% of female cancer cases [33]. Our estimates of 5246 incident male cases and 9573 female cases relate to 2.1% and 4.2%, respectively. Moreover, in the Western world, the incidence in HPV-associated cancers in younger age-groups increases [22, 25, 34–36]. Successfully implemented HPV-vaccinations programs should reverse these trends during the next decades.

For long-term monitoring of time trends (over 2–4 decades), a focus on cancer sites with high HPV-attributable fractions in younger age-groups (35–49 years) might be reasonable, though effects can first be expected to become observable in the more distant future: In Germany, HPV vaccination commenced in 2007 for 12–17 year old girls. As the oldest vaccinated women from the birth cohort of 1990 will not reach age 35 until 2023, it will be 2038 before the 35–49 age-group consists entirely of potentially vaccinated women.

The attributable fraction method may however not be suitable for monitoring time trends in the incidence of HPV-attributable cancers for sites with low attributional fractions, such as vulvar cancer and the oropharyngeal cancers group. Using vulvar cancer as an example, 18% of all vulvar cancers diagnosed in 2013 were attributed to HPV infection; 82% were attributed to other causes. If HPV vaccination decreases the incidence of HPV-attributable vulvar cancer in future years, this will hardly become apparent in the overall estimates, and the effect of the vaccine especially in low-PAF cancers in absolute terms will be underestimated without regular monitoring of the prevalence of HPV DNA in cancer cells. A decreasing proportion of cancer with HPV-associated morphology at respective sites would be expected. The newly emerging clinical cancer registries in Germany (according to the Law on the Further Development of the Early Detection of Cancer and Quality Assurance Through Clinical Cancer Registries [37]) might provide another anchor for concomitant projects to continuously monitor the cancer incidence and HPV-prevalence in cancer for short and medium term effects from HPV-vaccination in Germany in the near future.

## Conclusions

Our estimates of the cancer burden at HPV-related sites along with the HPV-attributable fractions provide a baseline assessment of the HPV-related cancer burden in Germany and contribute to future evaluation of long-term HPV-vaccination effects on the population level. With population-based clinical cancer-registration, future monitoring of HPV-associated cancer in Germany is feasible with more accuracy and precision than using global estimates.

## Abbreviations

CI5: Cancer in five continents
DCO: Cancer cases notified from death certificate only
EU: European Union
HPV: Human papillomavirus
IARC: International Agency for Research on Cancer
ICD-O-3: International Classification of Diseases for Oncology
NOS: Not otherwise specified
OP: Oropharynx
PAF: Population attributable fraction
SCC: Squamous cell carcinoma
STIKO: German Standing Committee on Vaccination
UK: United Kingdom
US: United States of America
WHO: World Health Organization
ZfKD: German Centre for Cancer Registry Data at the Robert Koch Institute
